# A Meta-Analysis of Success Rates of One-Stage Versus Two-Stage Revisions in Knee Prosthetic Joint Infections

**DOI:** 10.7759/cureus.57533

**Published:** 2024-04-03

**Authors:** Devon Patel, Vanessa Shannon, Soumya Sharma, Jiayong Liu, Martin Skie

**Affiliations:** 1 Department of Medical Education, The University of Toledo College of Medicine and Life Sciences, Toledo, USA; 2 Department of Orthopedic Surgery, The University of Toledo Medical Center, Toledo, USA

**Keywords:** device associated infections, infection, knee, prosthetic joint infection, total knee arthroplasty

## Abstract

Prosthetic joint infections (PJIs) pose significant challenges following total joint arthroplasties and cause profound complications. They are associated with significant morbidity and mortality. One-stage revision involves the removal of the infected implant and simultaneous re-implantation of a new prosthesis in a single surgical procedure. The two-stage approach is traditionally more common in the United States and follows a deliberate sequence: the infected implant is first removed, followed by a period of antibiotic therapy, and then a second surgery for implant reinsertion. While two-stage revisions were traditionally considered the gold standard, recent advancements have introduced one-stage revisions as a viable alternative. One-stage revision offers the advantage of being a single procedure, significantly reducing the patient’s downtime without a functioning knee. Currently, there has not been a comprehensive exploration of the comparative outcomes between two-stage revisions and one-stage revisions. This systematic review and meta-analysis aimed to assess the outcomes of both one- and two-stage revisions for total knee arthroplasties (TKAs), by utilizing comparison studies as the foundation for analysis. Our search encompassed databases such as MEDLINE (Medical Literature Analysis and Retrieval System Online), Embase, and Cochrane to identify articles examining the comparative efficacy and outcomes of one- and two-stage revision procedures between January 2000 and June 2023. We employed keywords relevant to knee PJIs to identify comparative studies reporting on success rates, reinfection rates, microbiological findings, and other pertinent outcomes. Statistical analysis for this investigation was performed using Review Manager 5.4 (The Cochrane Collaboration, 2020) with a standard significance threshold set at a p-value less than .05. This meta-analysis incorporated six comparison articles and 802 patients. Two-stage revisions (547 patients) were associated with greater success rates (i.e., infection eradication) than one-stage revisions (255 patients) (p = .03). The studies did not suggest a difference in the microbiology of the infections. Two-stage revisions are associated with higher success rates than one-stage revisions in the treatment of knee PJIs. Future randomized controlled trials should evaluate the optimization of the management of these complications.

## Introduction and background

Total knee arthroplasty (TKA) has transformed the field of orthopedics, providing many patients with renewed mobility and improved quality of life [1]. It is projected that 3.48 million TKAs will be performed in 2030, which is a 673% increase from 2005 [2]. However, despite the remarkable success rates of TKA, prosthetic joint infections (PJIs) continue to be a topic of conversation within the realm of orthopedics and microbiology. PJI remains a formidable complication, posing significant challenges for both patients and healthcare professionals. PJIs can lead to pain, functional impairment, prolonged hospital stays, increased healthcare costs, and in severe cases, necessitate implant revision, arthrodesis, or amputation [1,3,4]. Two distinct approaches for managing PJIs have emerged as primary strategies: two-stage revision and one-stage revision.

The two-stage approach is traditionally more common in the United States and follows a deliberate sequence: the infected implant is first removed, followed by a period of antibiotic therapy, and then a second surgery for implant reinsertion [1,5]. One-stage revision involves the removal of the infected implant and simultaneous re-implantation of a new prosthesis in a single surgical procedure. Advantages of one-stage revision are that it is only a single procedure and reduces the patient's time without a functioning knee. The choice between these two strategies has had varying opinions among orthopedic surgeons and infectious disease specialists regarding their efficacy and long-term outcomes [1,3-6]. Furthermore, understanding the microbiology of PJIs is essential in devising effective treatment and prevention strategies. PJIs are typically polymicrobial with a complex interplay of various microbes. Identifying the specific pathogens responsible for these infections and understanding their unique virulence factors, antibiotic resistance mechanisms, and biofilm-forming capabilities are pivotal for tailoring treatment regimens [1,3].

A previous meta-analysis with aggregated data showed there is no difference in re-infection rates between one-stage and two-stage revisions [7]. Smaller studies also suggest there is no difference in patient-reported outcomes and Knee Society functional scores [8,9]. However, two-stage revisions are preferred by members of the American Association of Hip and Knee Surgeons (AAHKS) when treating PJIs [10].

Currently, there has not been a comprehensive exploration of the comparative outcomes between two-stage revisions and one-stage revisions. This systematic review and meta-analysis aimed to assess the outcomes of both one- and two-stage revisions for total knee arthroplasties (TKAs), by utilizing comparison studies as the foundation for analysis.

## Review

Materials and methods

Systematic Search and Study Selection

A literature search was performed using MEDLINE (Medical Literature Analysis and Retrieval System Online, Embase, and Cochrane databases. The search strategy included permutations of “total knee arthroplasty,” “prosthetic joint infection,” “comparison study,” “one-stage,” and “two-stage” (Table 1). Medical Subject Headings (MeSH) were used when possible. Two authors independently examined the results of the literature search to decide which publications to include. If there was disagreement, a third author was consulted. Inclusion criteria were publications between January 1, 2000, and June 1, 2023, that were in English. Exclusion criteria included case reports, editorials and comments, and protocol papers. Reference lists of articles were also examined to identify studies for inclusion. The Newcastle-Ottawa Scale was used to assess the included publications and the scores were converted to descriptors of “good,” “fair,” and “poor” according to the Agency for Healthcare Research and Quality (AHRQ) [11].

**Table 1:** Databases and Search Strategies.

Database	Results	Search Strategy
MEDLINE	51	(("arthroplasty, replacement, knee"[MeSH Terms]) AND ("infections"[MeSH Terms] OR "reinfection"[MeSH Terms] OR "joint infection"[Title/Abstract] OR ("prosthetic joint infection"[Title/Abstract] OR "prosthetic infection"[Title/Abstract] OR "prosthesis infection"[Title/Abstract] OR "prosthesis related infection"[Title/Abstract]) OR ("periprosthetic joint infection"[Title/Abstract] OR "peri prosthetic joint infection"[Title/Abstract] OR ("Periprosthesis"[All Fields] AND "infection"[Title/Abstract]) OR ("Peri-prosthesis"[All Fields] AND "infection"[Title/Abstract])) OR ("implant infection"[Title/Abstract] OR "implant related infection"[Title/Abstract]) OR ("arthroplasty infection"[Title/Abstract] OR "arthroplasty related infection"[Title/Abstract])) AND (("one-stage"[Title/Abstract] OR "one-stage"[Title/Abstract] OR "1-stage"[Title/Abstract] OR "single-stage"[Title/Abstract] OR "single-stage"[Title/Abstract]) AND ("two-stage"[Title/Abstract] OR "two-stage"[Title/Abstract] OR "2-stage"[Title/Abstract] OR "2-stage"[Title/Abstract]))) AND (2000/1/1:2023/6[pdat]) AND (english[Filter]) NOT ("Editorial"[Publication Type] OR "Comment"[Publication Type] OR "Letter"[Publication Type] OR "Review"[Publication Type]) AND ("Cohort Studies"[Mesh] OR "Randomized Controlled Trial" [Publication Type])
Embase	30	('prosthetic joint infection'/exp OR 'prosthetic joint infection' OR (prosthetic AND ('joint'/exp OR joint) AND ('infection'/exp OR infection))) AND ('one stage':ab,ti OR 'one-stage revision') AND ('two stage':ab,ti OR 'two-stage revision') AND 'knee arthroplasty' AND [english]/lim AND 2000-2023/py AND 'article'/it
Cochrane	8	MeSH descriptor: [Arthroplasty, Replacement, Knee] explode all trees and (periprosthetic joint infection OR prosthetic joint infection) and (single-stage OR one-stage OR one stage OR single stage) and (two-stage OR two stage), Filters: 2000-2023, English

Data Collection and Outcome Measurement

A standardized spreadsheet was used to collect the data. The primary outcome measurements collected were success rates, re-infection rates, and infection eradication rates. Data were homologated such that re-infection rates were converted to success rates (i.e., a re-infection rate of 10% equates to a success rate of 90%). Other outcome measurements included functional outcome scores and microbiology profiles.

Statistical Analysis

The data was analyzed using Review Manager (RevMan Version 5.4.; The Cochrane Collaboration, 2020). Forest plots with the odds ratios using a fixed effects model were created using RevMan. A standard p-value of ≤ .05 was used to determine statistical significance.

Results

**Figure 1:**
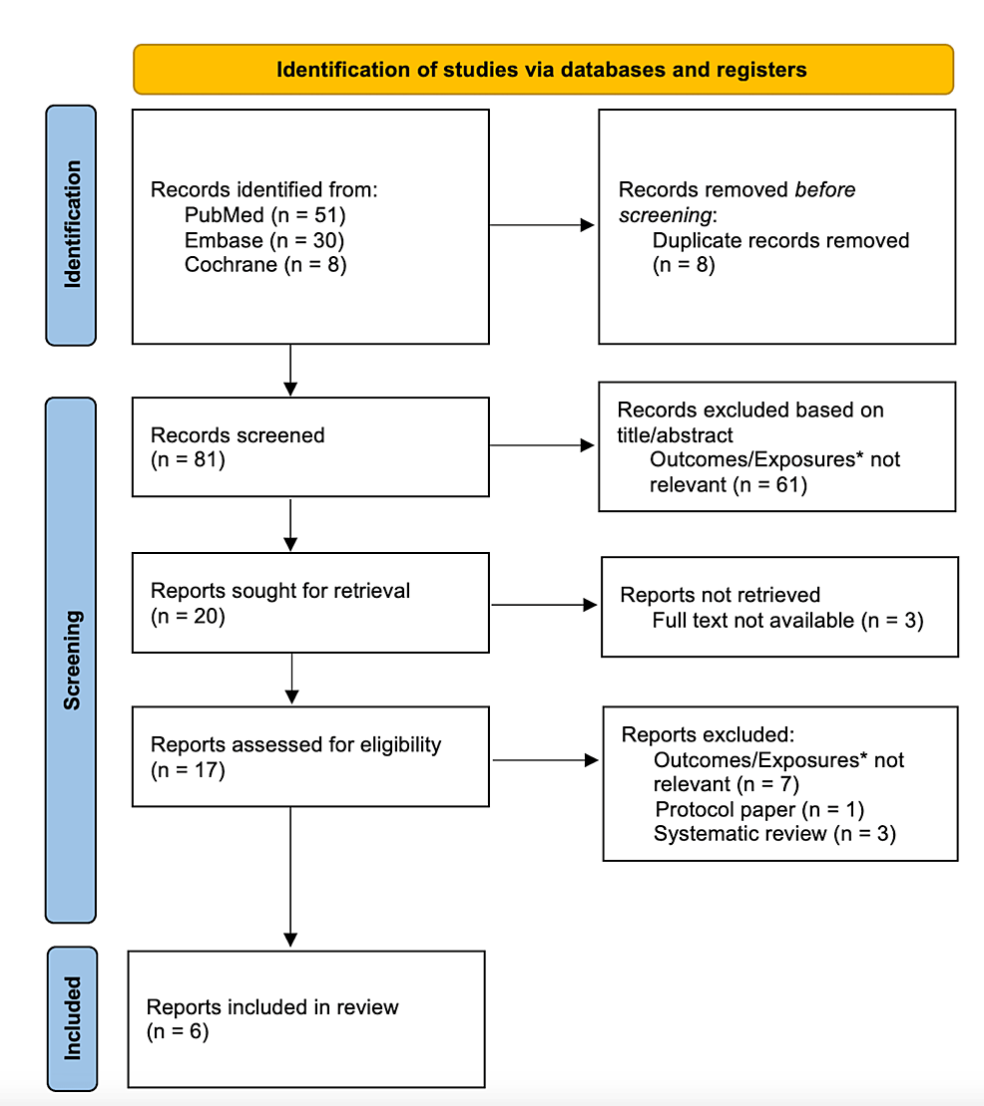
Flow chart demonstrating literature search and included studies. Flow chart demonstrating literature search and included studies. *Not relevant outcomes/exposures include reporting only one-stage or two-stage procedures, only one type of bacteria or fungus, comparisons of aseptic vs septic revisions, a unicompartmental arthroplasty, incorporated data about joints other than the knee without subset analysis, or comparisons that are not one-stage versus two-stage procedures.

**Figure 2:**
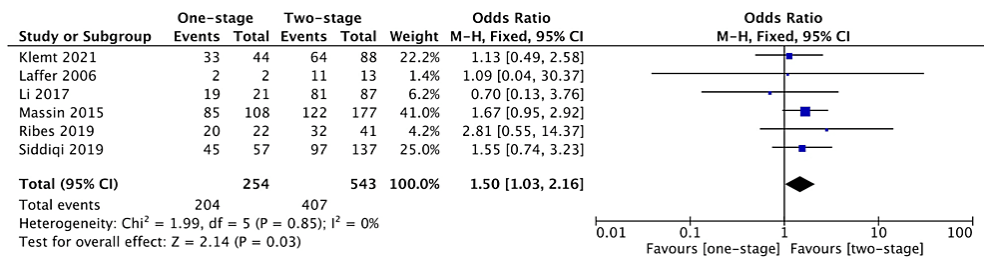
Forest Plots Depicting Outcomes for Comparison of One- and Two-Stage Revisions for Prosthetic Joint Infections After Total Knee Arthroplasty. References: [1,3-5,12,13]

Six articles reported success rates and rates of re-infection comparing one-stage (255 patients) and two-stage revisions (547 patients) (Figure 1) [1,3-5,12,13]. According to the Newcastle-Ottawa scale and AHRQ score conversion, five articles were “good,” and one article was “fair” (Table 2). There was a statistically significant difference in the odds ratio suggesting that two-stage revisions are associated with greater success rates compared to one-stage revisions (OR = 1.49, 95% CI [1.04-2.16], p = .03) (Figure 2). There was no significant heterogeneity between the studies included (I2 = 0%, p = .92). Table 3 shows the included studies and the outcomes measured.

**Table 2:** Newcastle-Ottawa Score and AHRQ Assessment for Included Studies. AHRQ: Agency for Healthcare Research and Quality

Study ID	Category Scores	Quality
Klemt et al., 2021 [1]	Selection: 3, Comparability: 2, Outcome: 2	Good
Li et al., 2017 [3]	Selection: 4, Comparability: 1, Outcome: 3	Good
Ribes et al., 2019 [4]	Selection: 3, Comparability: 1, Outcome: 2	Good
Massin et al., 2015 [5]	Selection: 4, Comparability: 2, Outcome: 3	Good
Laffer et al., 2006 [12]	Selection: 2, Comparability: 1, Outcome: 2	Fair
Siddiqi et al., 2019 [13]	Selection: 4, Comparability: 2, Outcome: 3	Good

**Table 3:** All Studies Included and Outcomes Reported. PROMS: Patient-reported outcome measures; PROMIS: Patient-Reported Outcomes Measurement Information System; IR: Infection Recurrence; KOOS-PS: Knee disability and Osteoarthritis Outcome physical function, Score; Physical SF 10A: Physical function Short Form 10A; SF-12: Short Form-12; OR: Odds Ratio; ROM: Range of Motion; IKS: International Knee Society Bold text indicates statistical significance (p ≤ .05)

Study ID	Outcome Measure	One-Stage	Two-Stage
Klemt et al. [1]	Preoperative KOOS-PS	47.9 ± 14.1	45.6 ± 13.9
	Preoperative Physical SF 10A	37.5 ± 6.3	35.5 ± 6.7
	Preoperative PROMIS SF Physical	38.8 ± 7.3	36.5 ± 7.0
	Preoperative PROMIS SF Mental	44.1 ± 10.7	43.5 ± 8.7
	Postoperative KOOS-PS	62.2 ± 8.3	51.9 ± 6.3
	Postoperative Physical SF 10A	42.8 ± 7.8	38.1 ± 7.7
	Postoperative PROMIS SF Physical	44.8 ± 8.9	41.0 ± 7.6
	Postoperative PROMIS SF Mental	50.5 ± 9.4	47.1 ± 7.8
Li et al. [3]	IR (%)	9.09%	6.90%
	Patient Satisfaction Rate (%)	77.3%	85.1%
	Reasons for discomfort	Reinfection, pain, stiffness	Reinfection, pain, renal insufficiency, nerve injury, joint fusion, amputation
Ribes et al. [4]	Infection Cure Rate (%)	90%	78%
	Knee Flexion ROM (degrees)	101.6°	86.1°
	Knee Extension ROM (degrees)	-0.2°	-1.8°
	IKS Objective Score	87.9	78.6
	IKS Satisfaction	22.3	17.21
	IKS Expectations	7.88	7.32
	IKS Functional Activities	51.05	39.75
	KOOS Symptoms	73.52	57.52
	KOOS Pain	68.23	57.45
	KOOS Daily Living	61.87	48.20
	KOOS Sport and Recreation	10.00	8.75
	Quality of Life	41.18	27.90
	SF-12 Psychological	43.11	38.08
	SF-12 Physical	39.10	34.41
Massin et al. [5]	IR Male (OR)	1	Articulated: 0.3, Non-articulated: 1.1
	IR Female (OR)	1	Articulated: 3, Non-articulated: 5.9
	IR (Multivariate OR)	1	Articulated: 1.7, Non-articulated: 4.4
Laffer et al. [12]	Success Rate (%)	100%	85%
Siddiqi et al. [13]	Reinfection Rate (%)	14.00%	24.10%
	Reoperation Rate (%)	19.30%	27.70%
	Success Rate (%)	78.90%	70.80%
	Final Total Arc of Motion (degrees)	105.8°	101.8°

Discussion

PJIs are associated with higher mortality rates than non-infected arthroplasties [14]. Multiple treatment methods can be used depending on the extent of the infection, soft tissue viability, and patient characteristics, amongst other things. However, there has been significant controversy surrounding which method is best. The present study demonstrates that two-stage revisions have greater success rates than one-stage revisions in knee PJIs. This is an important topic to review and analyze because of the need for more research on these devastating complications. Furthermore, our previous study of hip PJIs suggests that one-stage revisions are more successful than two-stage, so additional research should be conducted to elucidate these differences [15].

In 2023, Duncan et al. conducted a cross-sectional survey to assess and understand preferences and practice patterns in managing PJIs following TKA. Their study, which surveyed current members of the AAHKS using a 32-question survey, collected responses from 844 out of 2,752 members. Notably, over 75% of respondents expressed a preference for performing a two-stage exchange arthroplasty [10]. These findings lay the groundwork for further exploration into the reasons behind the prevalent preference for two-stage procedures.

An earlier consensus article outlined specific criteria that serve as contraindications for one-stage revisions, including the need for a bone graft, inadequate debridement of infected tissues, compromised soft tissue viability, presence of difficult-to-treat microorganisms, and insensitivity of organisms to antibiotics mixed into bone cement [6]. These factors potentially contribute to the higher success rates observed with two-stage revisions, particularly in cases of more virulent infections [16]. Additionally, two-stage revisions are associated with greater functional impairment, and this could be attributed to the lack of comprehensive physiotherapy during the interval stage [17].

A study conducted by Klemt et al. compared the effectiveness of one-stage and two-stage procedures. The findings suggested that patients who underwent one-stage surgery generally had better patient-reported outcomes and lower reinfection rates [1]. However, it is important to note that the two-stage revision is typically recommended for cases characterized by heightened complexity and resistant or culture-negative infections, posing a higher risk to patients. This could explain why the one-stage procedure reported better outcomes.

Another study conducted by Ribes et al. discussed the functional outcomes and reinfection rates associated with both methods. They ultimately concluded that the one-stage method yielded superior outcomes [4]. Further investigation is needed to assess the benefits of the one-stage replacement procedure, especially in cases where two-stage replacement is commonly chosen, such as in cases involving infections caused by resistant or unidentified bacteria. Large, multicenter, prospective trials should be performed to identify the most successful procedure for different patient characteristics, including the type of bacteria and length of infection. Randomized controlled trials would be optimal to evaluate which approach is superior because of the controversial data [18].

Numerous factors can influence the success of one- or two-stage revisions and can bias one procedure over the other. For example, one-stage procedures could be favored for patients without a sinus tract or gram-positive bacteria [19,20]. One-stage revisions are associated with a greater quality of life and functional outcomes than two-stage [21-23]. Furthermore, one-stage revisions are associated with lower costs and shorter hospital stays [24-26]. However, there may be novel techniques for two-stage revisions that can influence the outcomes of the comparisons, so future studies should evaluate these differences [27]. It is also important to address the presence of selection bias when interpreting the data from various studies and reviews [28]. There may also be unmeasured variables, such as alcohol abuse, that influence the reported success rates [29]. Building on these considerations, future studies investigating a patient's microbiome, genetics, socioeconomic status, lifestyle factors, and susceptibility to PJI can provide a better understanding of treatment outcomes and lead to a more patient-centered approach to deciding on the best procedure. Understanding how these factors influence infection development, response to treatment, and long-term outcomes can help optimize treatment protocols and quality of care in the management of PJIs.

## Conclusions

Two-stage revisions are associated with greater success rates in treating knee PJIs than one-stage revisions. Future research should attempt to elucidate potential explanations for this difference. Furthermore, ongoing research is crucial to discern the nuanced factors influencing the preference for two-stage revisions, particularly considering recent studies highlighting the potential advantages of one-stage revisions in certain scenarios. Addressing these complexities will be instrumental in refining treatment guidelines and optimizing patient outcomes in the management of PJIs. Future research should study trends in patient characteristics or operation management that maximize success, such as specific infection characteristics that are associated with greater success rates after one-stage revisions compared to two-stage. Additionally, exploring innovative strategies for infection prevention and refining surgical techniques may further enhance the efficacy of both one- and two-stage revision approaches. This approach represents a step forward in improving the understanding and management of PJIs.

## References

[1] Klemt C, Tirumala V, Oganesyan R, Xiong L, van den Kieboom J, Kwon YM (2021). Single-stage revision of the infected total knee arthroplasty is associated with improved functional outcomes: a propensity score-matched cohort study. J Arthroplasty.

[2] Kurtz S, Ong K, Lau E, Mowat F, Halpern M (2007). Projections of primary and revision hip and knee arthroplasty in the United States from 2005 to 2030. J Bone Joint Surg Am.

[3] Li H, Ni M, Li X, Zhang Q, Li X, Chen J (2017). Two-stage revisions for culture-negative infected total knee arthroplasties: a five-year outcome in comparison with one-stage and two-stage revisions for culture-positive cases. J Orthop Sci.

[4] Ribes C, Masquefa T, Dutronc H, De Seynes C, Dupon M, Fabre T, Dauchy FA (2019). One-stage versus two-stage prosthesis replacement for prosthetic knee infections. Med Mal Infect.

[5] Massin P, Delory T, Lhotellier L (2016). Infection recurrence factors in one- and two-stage total knee prosthesis exchanges. Knee Surg Sports Traumatol Arthrosc.

[6] Leone S, Borrè S, Monforte Ad (2010). Consensus document on controversial issues in the diagnosis and treatment of prosthetic joint infections. Int J Infect Dis.

[7] Kunutsor SK, Whitehouse MR, Lenguerrand E, Blom AW, Beswick AD (2016). Re-infection outcomes following one- and two-stage surgical revision of infected knee prosthesis: a systematic review and meta-analysis. PLoS One.

[8] Budin M, Abuljadail S, Traverso G, Ekhtiari S, Gehrke T, Sommer R, Citak M (2022). Comparison of patient-reported outcomes measures and quality-adjusted life years following one- and two-stage septic knee exchange. Antibiotics (Basel).

[9] Bauer T, Piriou P, Lhotellier L (20061). Results of reimplantation for infected total knee arthroplasty: 107 cases. Revue de Chirurgie Orthopedique et Reparatrice de L'appareil Moteur.

[10] Duncan ST, Schwarzkopf R, Seyler TM, Landy DC (2023). The practice patterns of American Association of Hip and Knee Surgeons for the management of chronic periprosthetic joint infection after total knee arthroplasty. J Arthroplasty.

[11] Wells GA, Shea B, O'Connell D (2014). The Newcastle-Ottawa Scale (NOS) for assessing the quality of nonrandomised studies in meta-analyses. https://www.ohri.ca/programs/clinical_epidemiology/oxford.asp.

[12] Laffer RR, Graber P, Ochsner PE, Zimmerli W (2006). Outcome of prosthetic knee-associated infection: evaluation of 40 consecutive episodes at a single centre. Clin Microbiol Infect.

[13] Siddiqi A, Nace J, George NE (2019). Primary total knee arthroplasty implants as functional prosthetic spacers for definitive management of periprosthetic joint infection: a multicenter study. J Arthroplasty.

[14] Thompson O, W-Dahl A, Stefánsdóttir A (2022). Increased short- and long-term mortality amongst patients with early periprosthetic knee joint infection. BMC Musculoskelet Disord.

[15] Patel D, Sparks A, Blood D (2023). Outcomes of 1-stage versus 2-stage revisions after hip prosthetic joint infection: a systematic review and meta-analysis based on comparison studies. J Orthop Physician Assist.

[16] Palmer JR, Pannu TS, Villa JM, Manrique J, Riesgo AM, Higuera CA (2020). The treatment of periprosthetic joint infection: safety and efficacy of two stage versus one stage exchange arthroplasty. Expert Rev Med Devices.

[17] Bernard L, Hoffmeyer P, Assal M, Vaudaux P, Schrenzel J, Lew D (2004). Trends in the treatment of orthopaedic prosthetic infections. J Antimicrob Chemother.

[18] Castellani L, Daneman N, Mubareka S, Jenkinson R (2017). Factors associated with choice and success of one- versus two-stage revision arthroplasty for infected hip and knee prostheses. HSS J.

[19] Silva Silva, M M, Tharani Tharani, R R, Schmalzried Schmalzried, TP TP (2002). Results of direct exchange or debridement of the infected total knee arthroplasty. Clin Orthop Relat Res.

[20] Matar HE, Bloch BV, Snape SE, James PJ (2021). Outcomes of single- and two-stage revision total knee arthroplasty for chronic periprosthetic joint infection : long-term outcomes of changing clinical practice in a specialist centre. Bone Joint J.

[21] Srivastava K, Bozic KJ, Silverton C, Nelson AJ, Makhni EC, Davis JJ (2019). Reconsidering strategies for managing chronic periprosthetic joint infection in total knee arthroplasty: using decision analytics to find the optimal strategy between one-stage and two-stage total knee revision. J Bone Joint Surg Am.

[22] Haddad FS, Sukeik M, Alazzawi S (2015). Is single-stage revision according to a strict protocol effective in treatment of chronic knee arthroplasty infections?. Clin Orthop Relat Res.

[23] Nagra NS, Hamilton TW, Ganatra S, Murray DW, Pandit H (2016). One-stage versus two-stage exchange arthroplasty for infected total knee arthroplasty: a systematic review. Knee Surg Sports Traumatol Arthrosc.

[24] Gehrke T, Zahar A, Kendoff D (2013). One-stage exchange: it all began here. Bone Joint J.

[25] Gehrke T, Alijanipour P, Parvizi J (2015). The management of an infected total knee arthroplasty. Bone Joint J.

[26] Wignadasan W, Ibrahim M, Haddad FS (2023). One- or two-stage reimplantation for infected total knee prosthesis?. Orthop Traumatol Surg Res.

[27] Iorio R, Iannotti F, Previ L (2023). A modified technique for two-stage revision in knee PJI treatment. J Clin Med.

[28] Lazic I, Scheele C, Pohlig F, von Eisenhart-Rothe R, Suren C (2021). Treatment options in PJI - is two-stage still gold standard?. J Orthop.

[29] Faschingbauer M, Bieger R, Kappe T, Weiner C, Freitag T, Reichel H (2020). Difficult to treat: are there organism-dependent differences and overall risk factors in success rates for two-stage knee revision?. Arch Orthop Trauma Surg.

